# Late Tying of the Umbilical Cord Gives the Child More Blood

**Published:** 1884-02

**Authors:** 


### Late Tying of the Umbilical Cord Gives the Child More Blood.

Dr. Edward Alcorn gives a resumé of the experience of various writers on this subject, which proves that the children whose cords are left uncut until after the placenta has been expelled thrive much better than those in whom it is cut before.—*N. Y. Med. Times.*

